# Sport-related concussion disclosure in women's rugby—A social identity approach

**DOI:** 10.3389/fspor.2023.1058305

**Published:** 2023-04-05

**Authors:** Lisa Ryan, Ed Daly, Alexander D. Blackett

**Affiliations:** ^1^ Department of Sport, Exercise and Nutrition, Atlantic Technological University, Galway, Ireland; ^2^ Department of Sport and Exercise, School of Health Science & Wellbeing, Staffordshire University, Stoke-on-Trent, United Kingdom

**Keywords:** women's rugby, sport-related concussion, social identity, brain injury, rugby union

## Abstract

**Introduction:**

Sport-related concussion (SRC) is a risk of collision sports such as women's rugby. To support appropriate SRC diagnosis and management, an understanding of the factors that encourage SRC disclosure is necessary. To date, research has focused on ascertaining individual player knowledge and attitudes towards SRC disclosure.

**Methods:**

We chose to investigate the potential influence of group identification effects by examining the role of social identity on SRC disclosure in elite women's rugby. Seventeen elite players from the United Kingdom and Ireland were interviewed and their transcripts thematically analysed.

**Results:**

The data highlighted that the players shared a very strong social identity as women in rugby and were acutely aware that their experiences were different to their male counterparts. The shared social identity had both positive and negative implications for SRC disclosure. The players interviewed did not feel comfortable disclosing their symptoms to their coach and often felt that medical staff either did not listen to them or were unavailable to them. Mediators such as communication, trust in medical teams, perceived pressure, positive injury management experience, and player role models were identified.

**Discussion:**

This research could be utilised to inform sport psychology interventions to enhance SRC disclosure in elite women's rugby.

## 1. Introduction

Women's rugby union (“rugby”) participation has been steadily increasing over the last number of years with females now representing over a quarter of the current playing population globally (1). In July 2022, the IRFU (Irish Rugby Football Union) became the last of the six nation countries (Ireland, England, Scotland, Wales, Italy, and France) to announce that they would be offering contracts to their women's 15s players (though specific details are yet to be released). This marks the beginning of a very different landscape in women's rugby and means that future generations may be able to view rugby as a legitimate career option. This will also undoubtedly mean that we can expect to see the recent increases in female participation in rugby union continue and exceed current projections.

The success in beginning to close the gap in gender participation in rugby is to be commended; however, sports science research has traditionally been male-focused making it difficult to establish evidence-based criteria for performance and injury management in female athletes (2). Females (in some countries) are now beginning to commence rugby in as early as 5 years of age and thus follow an early specialisation pathway into the game. It is the responsibility of all key stakeholders involved in the rugby union to ensure that increased female participation does not come at an increased risk to the wellbeing of the players. Strategies to prioritise athlete health and reduce the risk of serious injury, such as sport-related concussions (SRCs) (3, 4), must be addressed to avoid a concurrent increase in risk of injury, as participation in women's rugby increases.

Concussion in women's rugby is grossly under-researched despite the known risks. Concussion is a brain injury that occurs after force is applied either directly or indirectly to the head (skull) leading to the rapid acceleration and deceleration of the brain (5–7). The terms concussion and mild traumatic brain injury (mTBI) are sometimes used interchangeably (8), though there are some distinct and subtle differences (9–11). A clear and agreed definition of SRC is essential to ensure that appropriate diagnostic and management guidelines are developed and adhered to. The definition developed by the Concussion in Sport Group (CISG) has been widely adopted by a variety of sporting bodies, including women's rugby (12). The CISG defines an SRC as a “mild traumatic brain injury (mTBI) induced by biomechanical forces transmitted either by a direct blow to the head or elsewhere in the body whereby an impulsive force can be transmitted toward the head” (11). The Sport Concussion Assessment Tool (SCAT), also developed by the CISG originally in 2004 (13), was used to provide a standardised tool to assist in diagnosing SRC in a field-based/sideline setting. Research has highlighted however that the management of concussion in sport is not standardised exposing players to potential risk in the management of their symptoms (14).

Collision sports, such as rugby, represent a high degree of risk for SRCs. A systematic review by King et al. (3) highlighted that the incidence rate of SRC was 2.8 per 1,000 h of match play for women's rugby-15s and 8.9 per 1,000 h of match play for women's rugby-7s. Table 1 highlights the available data for the injury rate and incidence of SRC in women's rugby. While there are growing concerns about the long-term effects of SRC, particularly related to repetitive head injury, experiencing an isolated SRC may not in itself pose a health risk if managed appropriately. There are a number of steps that can be taken to mitigate against potential health effects such as immediately removing players from play if an SRC is suspected, using a validated tool such as the SCAT5 to provide a diagnosis, and following an appropriate return to play (RTP) protocol (11). World Rugby (23) has recently changed the “graduated return to play (RTP)” protocol (referred to as the “accelerated RTP” by players) for elite rugby players from the previous 6 to 12 days though there are calls for this to be extended to 21–28 days based on current available evidence (24). A recent study by Madhok et al. (25) investigating the recovery outcomes in patients diagnosed with mTBI [with a Glasgow Coma Scale (GCS) of 15 and a negative CT scan] found that 56% of participants had incomplete recovery after 6 months with female patients more likely than male patients to have incomplete recovery [Relative Risk (RR), 1.31; 95% CI, 1.15–1.49]. This may have implications for SRC management as, while many players who sustain an SRC are reported to recover within 1 month of injury, between 6% and 28% report symptoms that persist beyond this timeframe (26–28).

**Table 1:** SRC injury rates and incidence in women's rugby.

Author (Year)	Participants	Injury rates/SRC incidence
Kerr et al. (2008) (15)	Female collegiate rugby union players	SRC injury rate of 1.58 per 1,000 player-game hours (*n* = 25) for matches. SRC injury rate of 0.30 per 1,000 AEs (*n* = 7) for training
Peck et al. (2013) (16)	Female collegiate rugby union players (*n* = 129)	SRC injury rate of 4.37 per 10,000 AE (*n* = 30) s
Armstrong and Greig (2018) (17)	Female collegiate rugby union players (*n* = 64)	9% (*n* = 3) of female injuries were SRC
Fuller and Taylor (2020) (18)	Female international rugby-7 s players (*n* = 1,562)	15.6% of injuries were SRCs
Lopez et al. (2020) (19)	Female under-19 rugby-7s teams (*n* = 61)	14.3% of injuries were SRCs SRC injury rate of 22.2%
King et al. (2020) (20)	Female amateur rugby union players (*n* = 69)	SRC injury rate of 0.3 per 1,000 training hours (*n* = 1) SRC injury rate of 16.1 per 1,000 match hours (*n* = 9)
Teahan et al. (2021) (21)	Female collegiate rugby players (*n* = 30)	SRC injury rate of 3.7 per 1,000 match hours Burden of SRC injury: 27.9 days absent/1,000 h
Yeomans et al. (2021) (22)	Female amateur rugby union players (*n* = 234)	5.5 SRCs per 1,000 player-hours Burden of SRC injury: Median of 21 days missed per SRC.
King et al. (2022) (3)	Female rugby union players (pooled analysis)	SRC injury rate of 2.8 per 1,000 match hours in rugby-15s SRC injury rate of 8.9 per 1,000 match hours in rugby-7s

AE, athletic exposure; SRC, sport-related concussion.

There is considerable growing concern over the potential long-term consequences of repetitive head injuries (as may be seen with multiple concussions) (10, 29). A distinct neuropathology termed chronic traumatic encephalopathy (CTE) has been retrospectively linked with repeated head injuries (30). CTE was originally observed in boxers (labelled as “punch-drunk”) and has since been retrospectively diagnosed in American and Australian-rules Football, rugby, soccer, and ice hockey among other sports (30). Nowinski et al. (31) applied the Bradford Hill Criteria for causation to the available data and have suggested for the first time that there is a causal link between repetitive head trauma and CTE. A number of other neurological disorders have been associated with mTBI and repetitive head injuries including mood disorders, sleep impairment, depression, cognitive impairment, and neurodegenerative disorders (10, 29).

Though changing, a considerable amount of SRC literature has focused on male athletes. Research has suggested, however, that female athletes may be at greater risk of sustaining an SRC, may take longer to recover, and may have more severe symptomology than their male counterparts (32–34). Symptoms also tend to present differently between the sexes; female head injuries, for example, are more likely to lead to persistent headaches, mental fatigue, anxiety, difficulties in concentration, and mood changes. Female athletes also seem to experience their symptoms for longer than their male counterparts (33). Further evidence is required to elucidate whether females truly experience concussion different from male athletes or whether underlying factors such as differences in support structures and a lack of medical support in many female sports may play a role rather than physiological differences. One proposed explanation may be that female athletes are more likely to self-report/disclose an SRC (32, 35, 36) compared to their male counterparts.

Six studies have retrospectively examined the association between gender and SRC non-disclosure (37–42) with contradictory findings. Two out of the six studies examining gender and SRC non-disclosure reported that male athletes were less likely to disclose suspected SRCs than female athletes (37, 40), while the remaining four studies did not report any differences between genders (38, 39, 41, 42). Interestingly, a history of concussion has been found to play a role in SRC non-disclosure (37, 39, 43), and this could be linked to negative player experiences in the injury management process post-SRC diagnosis reducing the likelihood of future disclosures. Understanding the variety of factors that may influence SRC non-disclosure behaviour is important as it may help inform targeted intervention strategies aimed at improving SRC disclosure (36). To date, much research has focused on the individual athlete and their motivations, knowledge, attitudes, and behaviour (44); few studies have considered how the group, such as a women's rugby team, interacts and develops a shared identity, which may impact on SRC disclosure and injury management strategies.

### 1.2. Theoretical framework—a social identity approach to injury management and SRC disclosure

Though still relatively new to the area of sport and exercise psychology, the social identity approach (SIA) has increasingly been utilised to investigate and explain group behaviour in sport and exercise settings. The theory has arisen from social identity (SI) theory (45) and self-categorisation theory (46) and places emphasis on understanding group processes and the psychology behind them. SI is the process through which individuals stop thinking in terms of “I” and “me” and instead think in terms of the group as “we” and “us.” When individuals begin to think in terms of the group rather than themselves, it can influence a number of different facets such as their behaviours (47–49), beliefs (50), stress management (51–53), cognitions (54), and wellbeing (55, 56).

Individuals thinking in terms of their team (team identity) rather than themselves (personal identity) is generally seen as positive (and indeed desirable) in sports settings and thought to be beneficial for sporting performance (57). Consensus has not been reached as to what motivates individuals to identify as a group; however, Thomas et al. (58) proposed different factors that may predict group identification. Their research across 369 participants involved in 45 different sports across two countries (England and Italy) highlighted that four personal identity motives (a person's individual feelings of self-esteem, distinctiveness, and meaning and efficacy resulting from membership of the team), three social identity motives (a person's individual feelings that the team identity conveys a sense of meaning, belonging, and continuity), and one collective identity motive (a collective belief in group distinctiveness) were predictive of group identification.

Many sports psychology interventions aimed at enhancing cohesiveness among team members are often trying to develop the shared identity of the team so that players will begin to think and function as one. Such an identity on a sporting field has many benefits as members can grow to instinctively understand the movements of their players, their motivations for different passes/phases of play, and enhance the confidence of the team (59, 60). While a considerable amount of research has focused on the potential beneficial effects of social identity on sporting performance and sports leadership (61–63), there is also evidence to suggest that there are instances where social identity can be a basis to behaviours that are harmful to health. Monaghan et al. (64) found that bodybuilders’ shared social identity could influence an individual's adherence to particular codes of conduct and lead to acceptance of drug-taking. A shared social identity, therefore, may influence an individual's view of social norms that are shaped by the group. It is the latter observation that serves as the motivation for the current research. Given the increase in popularity and uptake of women's rugby and given the known risks of rugby on concussion and mTBI, our research questions were two-fold:

(1) What are the factors that influence women's rugby players’ disclosure of head injury or whether they continue playing while injured?
(2) To what extent can players’ actions be explained by social identity theory?

Previously investigated factors that influenced SRC disclosure in professional male rugby players (65, 66) highlighted financial pressure, contract negotiations, and peer influence as deterrents. Additionally, the language used by male players to describe their symptoms was different to that used by their medical teams (which may have implications for diagnosis and treatment). To date, no research has explored the factors that influence SRC and brain injury management in elite and semi-elite women's rugby.

The social identity approach to sport and exercise psychology has been heralded as a new era of psychology in the sport and exercise field. Its proponents argue that this approach can be used to enhance sporting performance. Few studies have investigated how social identity may influence injury management. Knowing that female rugby players may be at a greater risk of concussion than their male counterparts (67) means that understanding of the influencing factors and the environment surrounding concussion in women's rugby is essential to inform best practice for SRC management. We chose to analyse this area utilising the SIA to ascertain how players’ social identity may impact on their injury management strategies, especially their openness around SRC and head injury disclosure.

## 2. Methodology

The study's objective was to analyse how the social identity of women rugby union players in the United Kingdom and Ireland influences their management of injury, in particular, sports-related concussion. The ontological position of a “critical realist” was taken where it is acknowledged that while a reality exists independent of the observer, we cannot know that reality with certainty (68). This was accompanied with a constructivist epistemology in which meaning is created from the interplay between the subject and the object (the subject *constructs* reality of object). Semi-structured interviews (Appendix) were employed to enable participants to reflect and recall their personal experiences with concussion, physical injury, injury management, and concussion in depth (65, 69). This approach also enabled the researcher to explore themes with further probing questions. Interview questions were designed to elicit responses on participants’ playing background and experience in women's rugby and to establish their thoughts and opinions on physical injury and SRC experience. Participants were sought from the United Kingdom and Ireland to get comprehensive perspectives (due to differing set ups at the time of research collection, some international players were contracted, others were not).

### 2.1. Ethics and procedures

Ethical approval, according to the Declaration of Helsinki, was granted to this study *via* the university ethics committee. The initial cohort of participants was identified by the lead researcher. Invitations to participate were issued *via* email alongside a participant information sheet. Subsequently, preliminary discussions with participants took place to give participants the opportunity to discuss the aims of the study and how the information would be confidentially managed. Interview times and preferred methods for the interview were agreed at this point. Several methods for conducting interviews were offered to each participant, ranging from face-to-face, online (*via* Teams, Zoom, or Skype), or *via* telephone, and each participant notified the researcher of their preference (70). Each participant provided informed consent prior to their interview. It was established that all information would be treated confidentially and anonymised for the purposes of the study.

Interviews lasted between 29 and 68 min (*M* = 43.17 min, *SD = *12.1 min) in duration. All interviews were audio-recorded and transcribed verbatim by the lead researcher. Participants were asked if they would like to review their transcript. Ten out of the 17 participants received their interview transcript by email within 10 days of the interview to check for accuracy. There were no changes made to any transcripts.

### 2.2. Study participants

Participants for the study were selected through purposive sampling and accessed using snowball sampling. Three purposive sampling criteria were devised (71). These were as follows: (1) participants had to be women; (2) ≥18 years of age; and (3) playing elite or semi-elite rugby union in the United Kingdom and Ireland. A total of 17 participants were purposively sampled after confirming that they met all sampling criteria. All participants were international rugby union players representing Ireland or UK countries at the time the data were collected. Participants were also playing with the Premiership (Premier 15s, United Kingdom) or representing their province and playing in the AIL (All-Ireland League). The playing positions were separated into forwards (*n* = 11) and backs (*n* = 6). Out of the 17 players interviewed, 16 had experienced at least one concussive injury during their time playing rugby, and 10 players had experienced ≥5 concussions (Table 2).

**Table 2:** Interviewee characteristics.

Player	Age when started playing rugby	Dual sports	Introduced to rugby *via*	Previous concussion	No. of concussions (*via* recall)^a^	Position
P1	25	Yes	University (Friend)	Yes	5	Forward
P2	24	Yes	University (Friend)	No	1	Back
P3	19	Yes	University (Friend)	Yes^b^	1	Forward
P4	14	No	Local club	Yes	11–12	Back
P5	5	No	Family (Brothers)	Yes	6–7	Back
P6	19	Yes	University (Friend)	Yes	3–4	Forward
P7	18	Yes	University (Friend)	Yes	6–7	Forward
P8	19	Yes	University (Friend)	Yes	2–3	Forward
P9	19	Yes	University (Friend)	Yes	4–5	Forward
P10	21	Yes	University (Friend)	Yes	5	Forward
P11	N/D	Yes	University (Friend)	No	N/A	Forward
P12	7	No	Family (Sister)	Yes	5+	Back
P13	17	Yes	University (Friend)	Yes	1–2	Forward
P14	18	No	University (Friend)	Yes	5+	Forward
P15	5	No	Family (Brother)	Yes	3–4	Forward
P16	18	Yes	University (Friend)	Yes	6	Forward
P17	19	No	University (Friend)	Yes	2–3	Back

Dual sports, player was playing another sport when introduced to rugby; N/D, not disclosed.

^a^ Recall, those diagnosed and those suspected by player.

^b^ Not rugby related.

### 2.3. Researcher description

The lead researcher identified as a woman and has been involved in higher education teaching in the United Kingdom, Ireland, and/or Australia for 18 years. The lead researcher has been working with (predominantly male) rugby union teams for over 20 years and is the co-founder of the Irish Concussion Research Centre and a vocal advocate for the support of sportswomen. This backdrop underpinned the desire to investigate this issue. The lead researcher is an experienced qualitative researcher who has previously conducted similar interviews and analysis with male rugby players. The researcher's background was reflected upon before data collection through the process of bracketing to mitigate any potentially negative effects of preconceptions on the research process and to increase the rigour of the process (72, 73).

### 2.4. Data analysis and methodological integrity

Data were analysed deductively against a social identity framework and thematically utilising the reflexive thematic analysis approach developed by Braun and Clarke (74, 75). This six-phase guide to thematic analysis began with familiarisation with the data—transcribing the data from the recordings, rereading the interview transcripts, and noting any initial ideas on the data as a whole. Phase 2 generated initial codes, highlighting meaningful text from the transcripts, collating them into a new data set relevant to the research topic, and coding them based on the interesting features in the data. During data analysis, priority was offered to the “voices” of the participants and these were regarded as the “primary source of knowledge” (76); however, an awareness of our own interpretations was recognised and reflected upon throughout the process. Phase 3 searched for themes in the data, grouping all relevant data items relating to each potential theme. After phase 3, the project team (researchers and supervisor) met to evaluate the potential themes coming through. Phase 4 reviewed the themes and a map of the thematic analysis was produced to analyse how well the themes captured the coded extracts. Phase 5 further defined and refined these themes, identifying the specifics of each theme starting the overall analysis. The final phase was the complete analysis of the data extracts identified under each theme.

## 3. Results

The main aim of the present study was to explore how social identity may influence injury management in women's rugby union players, particularly in the case of sport-related concussion. Three key points emerged from the analysis [SIA tenets quoted from Rees et al. (77)]:

1. Women rugby players identify as women rugby players: *SIA tenet—Social identity is both relational and comparative*.
2. Social identity may have a positive effect on injury management when those within the “ingroup” have sufficient knowledge of the injury: *SIA tenet—Effective support from others generally depends on those others being representative of a shared social identity*.
3. Social identity may have a negative effect on injury management when players avoid disclosing injury or play injured due to their commitment to the team: *SIA tenet—People's willingness to sacrifice personal interests for those of the group is driven primarily by identification with the group*.

### 3.1. Women rugby players identify as women rugby players: social identity is both relational and comparative

**Figure 1:**
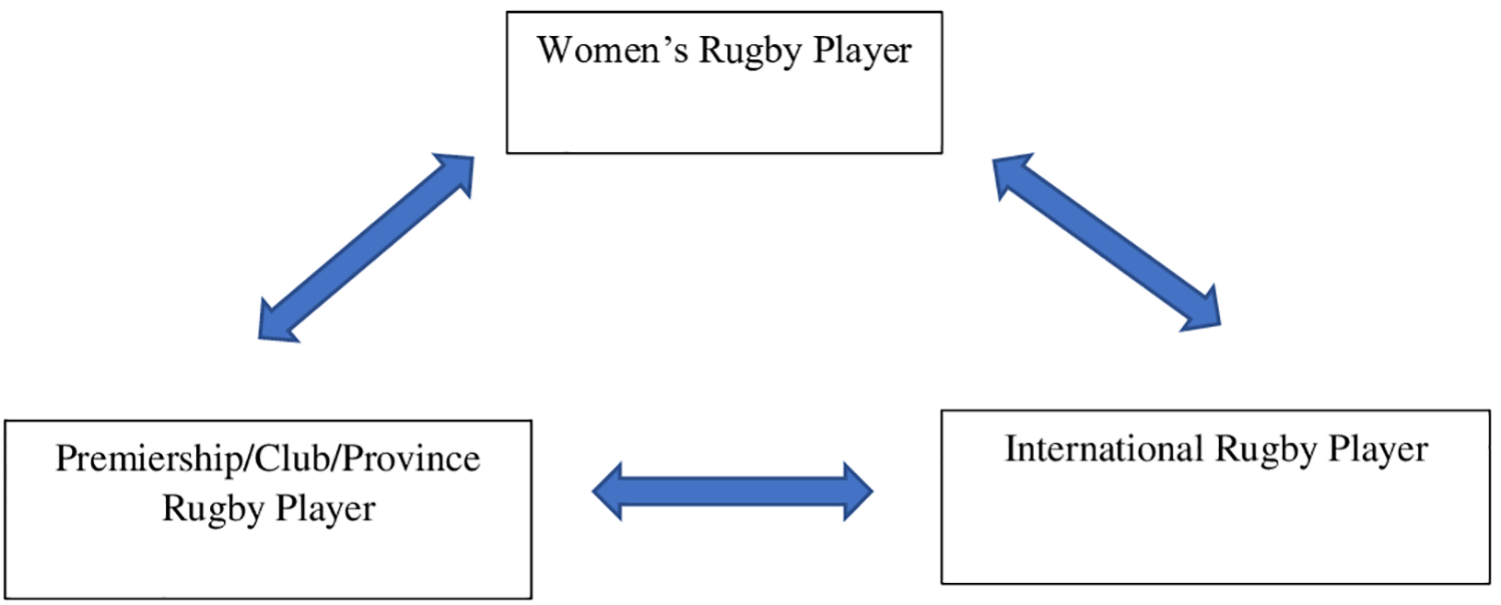
Player social identities (hierarchy).

Players clearly identified as women rugby players, as distinct from simply a rugby player (Figure 1). Many of the players interviewed had experienced negative comments about women in rugby. One player recounted an encounter with the parent of an ex (male) international rugby player.

And I remember meeting his dad where I worked. So, he was retired and we were kind of chatting rugby and he was like, “my wife believes that women shouldn’t play rugby” and I found this fascinating. I was like what? (P11)

Players spoke openly about differences between the men's game and the women's game such as inadequate strength and conditioning (S&C) support.

Maybe they're (support staff) just spread too thin, so I think a little bit of extra support would be the first and the men's team would have had S&C, they would always have had an S&C involved. … but definitely the women could hugely do with input from S&C I think just from an injury prevention starting from injury prevention before it even gets to that level. (P7)

Differences regarding nutritional support were made also: “Yeah. Because I think that's definitely a part that's sort of missing just in terms of well, nutritional guidance and everything isn't that great in the women's game anyway” (P9).

The women interviewed also noticed differences in how their sport-related injuries were managed compared to their male counterparts.

So the difference with the, the maybe the women being more time based, time-frame based whereas the men's (injury management) are landmark based. You know if you can hop or if you can you know if you can functionally achieve a landmark then you can progress to the next phase of your rehabilitation or or whatever it is. And whereas with women I find it's time. You know you must spend six weeks doing this and then you must spend … and I, [was thinking to myself] really six weeks?! ‘Cause I know someone who only spent two. (P1)

Like many athletes, players were willing to put up with a lot to play the sport they love.

Why would you do that and why would you go through all of these injuries? And was it worth it? And of course it was. You know I got to experience some of the best things that a sportswoman or or person could ever get to experience. Uhm, you know I still, I still have trouble with the injury that potentially was going to finish my career. (P5)

Players also acknowledged (frequently) that they felt “lucky” or “fortunate” to have set ups that are taken for granted in the men's game.

You know we have all of our games video properly and it sounds it sounds silly that of course you would get that at international level, but that's not the case necessarily with women's rugby. And yeah, so I think that I've always been very fortunate to be in a setup that has supported it. (P5)

Many of the women interviewed also believe that they have a responsibility to keep on striving for change in the women's game and advocating for the next generation of players.

So yeah, we've just look a long way to go and and I think you know yourself, you said there with the funding and like it's like if the loudest scream to to want to better standard. I'm like this is not an argument like it's nearly like an old school. Like if women you know, if women shout loud enough, we're nags or we're you know … we want something that we don't deserve and I'm like we're again we're caught between a rock and a hard place cause we want standards we again we have to. You know, you know, bide by the rules and be lovely particular women and wait for our opportunity and now we're still under the thumb and scrutinized, and it's it's it's painful. (P11)

And ensuring that there is adequate injury management and support at all levels,

The making sure that players understand the severity of it (brain injury) is really important and actually irrelevant of the level of the game, and I think that we get so tied up in the elite game that we've, we forget that we have to be insistent on it and that responsibility around grass roots and the developing side so that people understand and actually put in place support around people that don't have full time medics, don't have full-time strength and conditioning coaches don't have full-time coaches. (P5)

The fact that the women interviewed feel they have to continuously strive for improvements, face negative comments, are under resourced, under supported, and also feel a responsibility to pave the way for future players highlights that elite women's rugby players are contending with a notable number of issues typically not experienced by their male counterparts. The players interviewed clearly strongly identified as women in rugby, unified in their struggle for better support and recognition for the women's game, and this shared social identity seems to play a role in how they manage and discuss their injuries (in particular SRC/brain injury). The coaching staff, medical staff, and S&C staff were not viewed as part of the “ingroup,” which could play an important role in how injury recovery strategies and messages are received by players. The present study highlighted some unique aspects into the role of social identity on injury management.

### 3.2. Positive effects of SI on injury management: effective support from others generally depends on those other being representative of a shared social identity

Two major themes were identified when exploring the potential positive effect of SI on injury management: (1) players supporting players and (2) positive injury management. These were separated into subthemes (Table 3).

**Table 3:** Developed themes, subthemes, and example quotes: the positive effects of social identity on injury management.

Positive effect of social identity on injury management		
Theme	Subtheme	Example Quote
1. Players supporting players	*Players minding players*	As soon as anybody, like looks like they've knocked their head kind of people back off, or if anyone shouts like my head or everyone kind of … It doesn't matter where you are, you could be defending your line and everyone's like, stop, stop. So I think it is one, it's one of those ones that people seem to take quite seriously (P9).
		Player has has been on the borderline of being retired from rugby with the amount of concussion I've seen her being knocked out. And I can't remember the clinical term. You know, basically when she's sprawled near like she's Jesus on the cross and which is a very bad sign. You know, she's deep could cause. Erm And she tries to get up and play, you know? So I remember in a in a game saying, you know, ref, you need to stop, that 7 needs to stop. She's after being knocked out, you know. So you kind of. Yeah, you are aware it certainly be aware because it said I know myself I'd be trying to play on (P11).
	*Players learn from players*	To be honest. I think everyone's quite open with, you know, having conversations around their injuries. I don't know if you've met her before, but she had. She's went through quite a few injuries this season. Um and one of our injuries were quite similar and we getting the same symptoms so she was like giving me, I think it was a game ready or something like that to sort of help with swelling and pain and and sort of giving me advice and what sort of helped out and what didn't, etcetera, etcetera. And we like players that probably injured similar areas of their bodies around the similar amount of time. I think there must have been a point where four of us was in (P14).
2. Positive injury management	*Player role models*	It's sometimes it takes maybe someone who's experienced and symptoms … one of your (own) players to actually, you know, reiterate the importance of how … you know how dangerous concussion actually is (P1).
		Maybe seeing other players really struggling with return from concussion, that you kind of … that kind of resonates with you a little bit more than than what it would just with an online module and how we can actually impact your career or when you see somebody retire and because of of concussion, uh, I think maybe it's taken a little bit more serious but it definitely there's plenty of room for more awareness. (P17).
	*Listen to players*	And then before I did my actual return to like full running and contact um I think it was Amy^a^ that was in the club at the time. She was like look this is probably what you'll need to do. I just text her being like messaging her be like oh I'm doing really well this is what I've progressed (P15).
	*Culture of player honesty*	But by saying something as soon as you say the word concussion you know there's a whole process. And obviously I've missed the next game. but then I was like, Well, you've got to be honest. Your team and you've got to kind of have that level of honestly, with yourself and the coaches and stuff. So they were like. OK (P4).
		And the players, I think on the whole, ignoring the … my own story, players on the whole are very honest about symptoms. Umm, like if they've got a headache or something like we've had a girl that missed out on the last game of the season. But you know, technically she could have played as in there would have been enough time, but she knew herself that she still had symptoms from the concussion and she didn't play. Umm. Which I think. And she's a very good player. Like I think I think he would do take it much more seriously than perhaps they used to (P9).
	*Players listened to (by medical staff)*	To be honest. It depends. It actually … we've got we've got a new physio now (but) with the previous one, it kind of depended who you are and how much you knew maybe about your own body or about your own previous injuries or how to manage training or how to manage you know whether it was an important game coming up that weekend, it would really depend on the player and how confident they were and what was wrong number them. But that would also depend whether the physio would almost listen to yourself your self prescription (P10).

^a^ Name changed to maintain confidentiality.

#### 3.2.1. Theme 1: players supporting players

##### 3.2.1.1. Players minding players

Players spoke fondly about their “love” for the game which was credited to both rugby itself and the social interaction that it afforded. Player's social identity with their group (of fellow players) lends itself to players looking out for and protecting each other and calling out if they think someone is injured or at risk of injury (Table 3). Players seemed particularly aware of minding players they knew had a history of concussion.

I've already had my go and all the other players had as well and then someone had to go again. So [the players said] “let's put someone else out there”, like my own teammates. They were willing to go again, so they were willing to go like “now let's protect her” a little bit … which was just lovely and really nice. (P4, when coming back from a brain injury)

##### 3.2.1.2. Players learn from players

A recurring comment throughout the interviews with these women was their lack of regular access to medical support such as physiotherapy. As a result, players learned a considerable amount of their injury management from each other. Players spoke about how they would discuss injuries openly with each other and the rehabilitation plans they had been given (either currently or previously) by their respective medical teams. This sharing of knowledge from player to player seems to be a source of support when access to medical treatment is low. One player suggested that this knowledge should be available in an injury rehab protocol database that each player could access if they cannot get regular slots with the physiotherapist or tailored programmes from their (club) S&C staff (as otherwise players’ injury knowledge is limited to the knowledge of the group).

And I think just to have a body of information about like injury or sort of a generic … kind of database of, of injuries and generic rehab plans for them or like things that you can do to help prevent XY or Z like a generic ankle prehab or shoulder prehab or whatever it needs, or something like that, because I think that it's all a little bit disconnected at the moment, even with the concussions. Like if you happen to know somebody that knows that creatine might help, then you would take it. But otherwise, unless you happen to be having a conversation with a player that suggests it, you're not gonna be told and you wouldn't know. (P9)

#### 3.2.2. Theme 2: positive injury management

##### 3.2.2.1. Player role models

The strong shared social identity of these players means that injury information received from “outgroup” members is generally not adhered to. Several players spoke about the power of the player experience around head injury education and how listening to fellow players discuss their struggles with concussion resonated far more than any online education module.

And whereas when you then have a teammate who is experiencing difficulties or extended symptoms, I think it probably actually maybe takes that to realize that, you know God that's actually something that could be really really, debilitating from a training perspective and from a return to play perspective but also from a long term perspective and and it was. Yeah. (P1)

##### 3.2.2.2. Listen to players

Players learn injury management from each other but also listen to the advice they receive from each other. The player voice is, therefore, very important in injury management strategies (Table 3).

##### 3.2.2.3. Player honesty

While players are aware that it is “easier” to hide a brain injury than, for example, a broken leg, players all spoke about the culture of honesty that they felt prevailed both from themselves and their teammates.

I actually took myself off. And because I went in to make a tackle and I got hit in the hip or sorry, someone's hip hit me here in the temple. And I didn't fall to the ground, but I came out the other side and I was looking the other way and I couldn't really, couldn't really gather myself. I hadn't, you know, touched my head or anything. Nobody had noticed that it was, that I had head injury. But I walked straight off. I said I need to. I'm not OK. (P10)

##### 3.2.2.4. Players listened to (by medical staff)

Players have had a diverse range of experiences with medical staff. Trust is an important component of listening to and adhering to injury management guidance. A positive injury management environment is one where players feel that they are being listened to (and heard) and feel comfortable disclosing their symptoms.

But I felt having the doctor kind of “in charge of that” was definitely more like, you feel kind of … I felt like more safe, if that makes sense. ‘Cause you're like they're touching base. Checking your symptoms every day and … also let you know that it's OK is if you're not. If OK, you pass your HIA (head injury assessment). But you know, your symptoms could still deteriorate, and it may not be mean that you're not concussed. (P1)

### 3.3. Negative effects of SI on injury management: people's willingness to sacrifice personal interests for those of the group is driven primarily by identification with the group

There appears to be considerable tension between the positive effects of SI and the potential negative effects. Two main themes were identified that may suggest a negative effect of SI on injury management in women's rugby players: (1) commitment and (2) playing injured (Table 4).

**Table 4:** Developed themes, subthemes, and example quotes: the negative effects of social identity on injury management.

Negative effect of social identity on injury management		
Theme	Subtheme	Example quote
1. Commitment	*Sacrifice*	So for me, it's easier because I take public transport and I could do all of my extra bits whilst on the train, but for other them the other girls when they're driving, they're like, OK, quickly leave their desks and they have to drive to the rugby club. They realize there is not enough time to. Just the amount of hours that women that play at this level put in. And working full time and playing rugby at the level is a lot like I don't think people realize how much time that we have to put into it and how much energy like the. (P16) So there is good transport links, but for two years I didn't have a car and didn't drive. So I after school I'd get the bus to X and then like the tube from Y. So I'd I'd leave school. I could leave a bit earlier but like I'd leave it like 3, 2–3 ish and then wouldn't get home till like half 11. So it was long old long long time like 2 days, 2–3 days a week (P15).
	*Physical Toll*	Literally everything. Every season, there's something I pick up like I've had concussions, I've been concussed this season. I've hurt my ankle this season. I've got this. I tore my bicep this season as well, like (P14).
		A couple of calf strains, I'd say two or three calf strains. Um, just there, they'll be more through periods of high load rather than anything else like it wouldn't. It wouldn't have been a case of it popped in a game or it got touring in the game. It was more so like. A strain turned into a, you know, a a deeper strain because I was using it too much or, you know, I didn't take enough rest (P10).
	*Training while injured*	And for injury and that's through, S&C, that's through conditioning and and being fit and whatever, but also knowing what weaknesses and tweaks or bad habits that we might have in our patterns. There's, we might know them, but there's no conversation around how you actually fit that in. On top of all the other stuff you have to do. You know, like you have that gym program, but you have to do this sort of stuff and like you can't fit that in. I actually honestly think the hardest time of training is when you're injured because you're doing your rehab. You're doing your conditioning like bike stuff to keep on top of fitness. It's if you're if you've a lower limb injury or even an upper injury. And then you're also doing the actual gym program as well to to whatever extent you can. I find you're you’re training twice a day, every day to when you're injured (P8).
	*Exhausted*	I was like I was going ‘cause I like. I can't. I really. I'm so tired. I can't. I can't do this. And then it's like, no, no. Well, if you can just come to two training sessions and then the other coach will say, well, if you can just come to 2 training sessions. So every coach will want you just for two or just for one. But they'll all want you. So you're still. I did definitely feel pulled and dragged (P1). But it did definitely take a toll and I definitely I know it's very hard … at least a burnout and but I didn't think at that stage I think that's how at 25 or 26 and I definitely felt like like I was burning the candle at both ends and then it kind of affected you were just fatigued and general and concentration and and things like that (P16).
	*Overtraining*	Because I wanna get make the Irish team and I need to know what you you're you're doing so I can do that. So I was playing with two clubs and I was doing the Irish training program on top of it because I thought that's what I needed to do. So I was just completely over over over training (P8).
		But it was just like the management of it all was, you know, no one, none of my coaches, they all knew I was training with different teams, but no one ever kind of said right actually, you know what? Don't train tonight. I know you trained just yesterday or or whatever so. And I always thought more with better rather than less (P17).
2. Playing injured	*Pressure (Coach)*	I hit my head on the floor and I was like, man, that is sore. But then I just went down and then. Oh, I didn't really go down. The coach was like “you”. I was like my head hurts and then and she like “you OK?”. I was like, yeah, I'm fine. And then that was it (P2).
		Very, very unlike, supportive with when and it like expectations that they put on the player. ‘cause I think it's the performance expectations that get put on a player then influence how you report. Because if you're getting pressure to play or pressure to perform a certain level before you can play and there's a big game coming up. It's difficult because it's on it's so self driven in terms of explaining your symptoms? (P5)
	*Pressure (Team)*	So you know, they're not exposed to all of these courses about concussion and when to take somebody out of the game or And you know, they're all about, uh, we don't have numbers. Sure get in there. You'll be fine. You'll be fine. You be fine. And it, you know, it is an issue. And you are pressured. And the teams themselves will go. You'll be fine. You'll be grand. And you know, we want to play kind of thing (P3).
	*Pressure (Time)*	And where they're like, OK, right. You've hurt your knee or you've hurt your ankle. You got a week and a bit to sort yourself out sort of thing (P14).
	*Insufficient players*	Anyway, we are struggling for numbers regularly enough. It goes in, you know, it goes in in leaps and bounds depending on the season, but a lot of the time you will be struggling for numbers at certain times and sometimes there is that bit of an onus to try and play with, come back at that little bit too soon (P7).
	*Feeling isolated*	Yeah, I suppose I'm I'm a crier anyway, so I'm yeah, it was very emotional period of time. … I was in a different country from my my social group might just most stability where? My friends in the team and I couldn't even see see them and so. It was … support networks were taken away from you (P6).
	*Poor communication*	So you know and and I did account you do a sign in and you sign out and stuff and I would, you know had signed in to say that I rolled my ankle at the conditioning the next morning my Achilles was pinching. So I went off feet but like no one organized for a Physio to assess me. I went and did it myself. I went and played a game actually that weekend and just strapped, thinking that might do it. I survived match but like was absolutely like limping. (P5) I suppose it's it's a it's a. It's a grey area for us because there's this whole “contracts” thing and getting injured or with international camp and getting injured, with club. And then who covers it, if that makes sense. So like if I got injured in international camp, they look after everything, do my follow-ups set my programs, all that kind of stuff and then pass me over then to Club (P8).
	*Education*	I think like the generic stuff you know like we'll say the the module online, you just really you flick through really fast because you're just like try and answer the questions at the end. (P1)
		I don't think I knew enough about what concussion was then to know that I was. I knew that I was in pain ‘cause I broke my nose. Uhm, so you know, did I want to be removed? One? No, because I'm competitive and I wanted you know, and we get knocks and bangs and bumps and bruises in rugby. Was I misleading the medics when they asked me did I feel OK? I think it's it's hard to know whether are you OK, you know where the old school saying over you hurt? Or are you injured and I think that that's the problem because there is this mindset of if you're just hurt then you just gotta get on with it. And where is that grey area of where is an injury and where is the long-term effect of damage to this player? (P5)

#### 3.3.1. Theme 1: commitment

##### 3.3.1.1. Sacrifice

Players were willing to sacrifice a wide variety of aspects of their life because of rugby. Players missed out on career advancement opportunities, gave up other sports that they loved, and spent hours travelling to and from training sessions.

Uh, I suppose from a medical perspective that if you were to get injured playing for another team that you then could, I guess not be part of Ireland. So, it was a case that you couldn't play, you weren't allowed to play another sport. (P1)

##### 3.3.1.2. Physical toll

Playing elite rugby seems to have taken an immense physical toll on the players; many players cited a variety of different surgeries that they have already been through as well as lingering injuries and potential long-term issues “but alongside all of that I had six reconstruction surgeries for various parts of my body, which is pretty tough” (P5).

##### 3.3.1.3. Training while injured

Many players spoke about continuing to train while injured or coming back from injury (too) early. A reason cited for this was “feeling like an outsider” (P17) when injured and missing their teammates.

##### 3.3.1.4. Exhausted

Though things are currently in a state of flux among the six nation countries regarding contracts, a number of elite players interviewed were representing their country, playing for the Premiership and also in either full-time university or work. Players spoke about the level of tiredness and exhaustion and the “lack of recovery” time and needing people to check if they are ok.

And she was like, you're really pale. You look really tired. You don't look well. Is everything OK? And it was the first time when someone asked me. Like, are you OK? You're like, Oh my God. No! I'm wrecked! (P1)

##### 3.3.1.5. Over training

Due to the current setup in the women's game, the total training load is still often not regularly monitored particularly when players move between international camp and their respective clubs. This lack of oversight over their total workload creates an environment where many players end up overtraining (Table 4).

#### 3.3.2. Theme 2: playing injured

##### 3.3.2.1. Pressure

Players discussed a number of different types of pressure that they feel, which leads to them playing injured while continuing to suffer with concussive symptoms. This pressure can sometimes be overt or simply implied by the coach but also can come from fellow teammates.

Very, very unlike, supportive with when and it like expectations that they put on the player. ‘Cause I think it's the performance expectations that get put on a player then influence how you report (your concussion symptoms). (P17)

##### 3.3.2.2. Insufficient players

A unique aspect of the (elite) women's game (compared to the men's) is that there can be periods in the season where their team is struggling to field a team. This can create a strong feeling of obligation in (injured) players who do not want to let their teammates down.

I probably could get there and we would probably be fine again, I don't want to let my team down. We don't have the numbers, which is an odd thing for a premiership club to not have the numbers. (P10)

##### 3.3.2.3. Feeling isolated

Players feel highly connected to their teammates who provide them with support on a number of different levels. When players are injured, they feel socially isolated (Table 4) and, therefore, some players may return early from injury to avoid the feeling of isolation.

##### 3.3.2.4. Poor communication

Players’ injuries can sometimes be missed or else poorly communicated vertically (between international camps and club) and horizontally between the club medical team and the coach.

Communication isn't always brilliant, and you're not sure as a player whether you should be the the person communicating between the two or whether they should be doing it themselves and it can get a little bit complicated and political, I guess. (P9)

##### 3.3.2.5. Education

Many of the women interviewed stated that they do not have enough knowledge regarding concussion and brain injury and that further education would help in their decision-making (to not play injured).

I think I think I'd still think there's plenty more room for awareness around and the long-term effects of concussion. And you know when you see players retire, you know their retiring, they're gone from the game, but you don't really understand the full effects of it. (P1)

## 4. Discussion

The primary aim of this paper was to examine how elite women's rugby players’ social identity may influence their injury management, particularly in the area of disclosure of symptoms of SRC. The interviews highlighted that the players strongly identified as women in rugby and were acutely aware that they were different to and treated differently from their male counterparts. The shared social identity of these women's rugby players seems to have a very strong influence on their management and disclosure of head injury and ongoing symptomatology, which may have both positive and potentially negative consequences on their overall health and wellbeing.

The SIA posits that when people think of themselves in terms of their shared identity (in this instance as a women's rugby player), it impacts the way they interact and relate to those around them (45, 78, 79). This shared identity can also predispose players to view their ingroup members as reliable sources of information (80). The current research has highlighted that social support and a shared social identity may benefit injury management through the sharing of information and the trust players put in the information they receive from their teammates. Players help each other through injury by providing injury advice when “staff are stretched too thin,” speaking up when they observe concussive symptoms in their teammates and by acting as role models and creating a culture of honesty to speak up when symptoms are present. The strong bond and connection that players feel to each other could, however, also have a potential detrimental effect as players do not “want to let their teammates down” and also feel an obligation to their team to play when numbers are low, and their team may not be able to field a team without them.

Brain injury is often underreported or not disclosed. Unlike a broken arm or a torn hamstring, it is possible to hide the physical effects of concussion from medical and coaching staff and continue to play while injured (65). Substantial efforts have been made by sporting bodies to enhance education around the management of and the effects of repetitive head injury and the importance of SRC disclosure (81). However, we also know that a player's drive to play means that they may still continue to play while injured (82, 83). Given the growing knowledge base around the potential long-term effects of repetitive head injury (84, 85), it is essential that we understand the motivations and influences on a player/team to disclose their symptoms. In the present study, coaching staff and medical staff were not viewed as part of the player “ingroup,” which may have notable implications for how injury management protocols are received and interpreted by players. The players interviewed did not feel comfortable disclosing their symptoms to their coach and often felt that medical staff (physiotherapists in particular) either did not listen to them or were unavailable to them. The study did, however, highlight the very positive role that players can have on each other and the important role that players themselves may play in distributing education around concussion and brain injury management. Many players stated that they only began to recognise the true potential impact of concussive head injury when they saw the effects on fellow players. This may mean that, for women's rugby players, online concussion education programmes are insufficient to drive behaviour change in concussion management and that coaching staff and sports psychologists should consider the use of player role models (perceived as part of the player ingroup) to help in the delivery of concussion education messages.

### 4.1. Communication

Communication seemed to be a key mediator in concussion and injury management. Players communicate openly with each other and readily discuss their injuries and symptoms. They were, however, reluctant to discuss this with the coach, which one player (P12) described as “the gatekeeper to whether you play or not.” Players have also felt let down by staff previously and have been left “isolated and confused” when injured and unsure who was meant to be looking after them (from an injury perspective). The lack of trust that some of the players had in their coach and medical team (outgroup) seemed to increase their confidence and trust in their fellow teammates (ingroup). At present, there seem to be a number of areas where women's rugby clubs and international setups could improve in their communication both directly *to* the player and to each other *about* the player regarding their injury.

### 4.2. Moving towards a social identity approach to SRC disclosure

**Figure 2:**
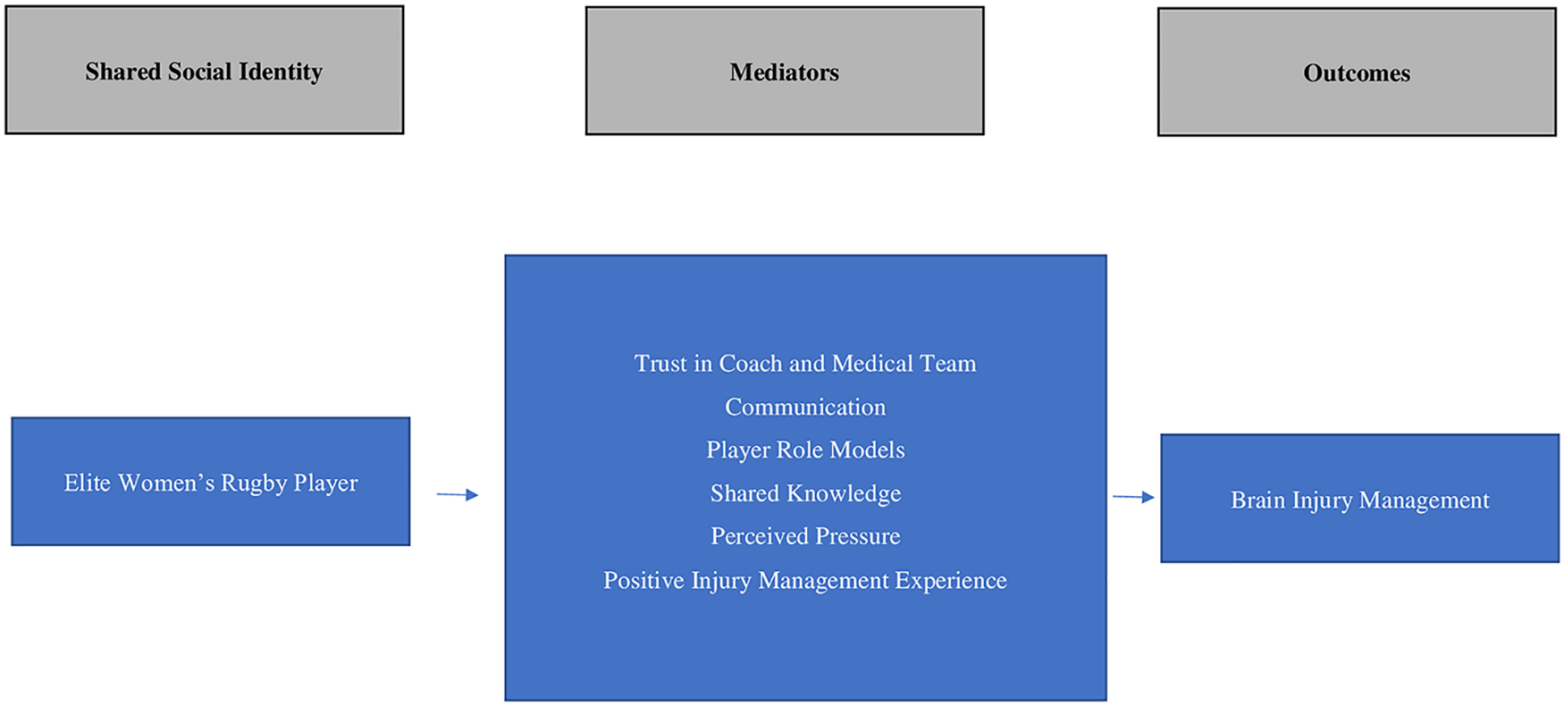
Proposed mediators of positive brain injury management and SRC disclosure. SRC, sport-related concussion.

To date, a considerable proportion of the research investigating players’ motivations for non-disclosure of a possible SRC has focused on SRC knowledge (38, 44, 86–89). This approach assumes that education is the main factor underlying appropriate SRC disclosure and management. However, there are a multitude of factors that may influence player behaviour and disclosure of injury (90). Previous research has shown that social support in sport is beneficial to overall health and wellbeing as well as enhancing psychological responses to injury (91). Social support may also be effective in supporting appropriate disclosure of injury. The present study has highlighted a new way of viewing and investigating player behaviour and suggested potential mediators (Figure 2) of SRC disclosure. It is important to move away from player-centric expectations of SRC disclosure and recognise some of the structural barriers that may prevent honest self-disclosure. Rather than focusing on interventions that target the individual in the disclosure of SRC, understanding the shared social identity of the group and targeting strategies that will be effective within that group may be more successful.

### 4.3. Applied implications

The current research emphasised the meaningful role that a medical, in particular a team sport, psychologist could play in enhancing disclosure of SRC and the subsequent management of SRC. Understanding the social identity of the group can lead to targeted strategies to improve communication. Many players interviewed are also studying for qualifications in medical fields or have previously qualified. Bringing players into the medical setups and coaching teams of rugby clubs could enhance trust between players and their organisations, which may improve communication and support for player health and wellbeing. The sport psychologist could also play a meaningful role in assisting players when they are injured with targeted management strategies to reduce feelings of isolation that lead to players returning (too) early from injury. It is important for sports psychologists working within women's rugby to consider how they position themselves and how they are seen by players to determine effective strategies for injury management and recovery.

### 4.4. Limitations and future directions

The themes identified through the current research are representative of a particular period in time. Women's rugby is changing rapidly and therefore a number of factors highlighted in the present research may not be applicable in the future. Though limited to a relatively small cohort of players (*n* = 17), this study has uniquely highlighted a number of important areas for sport psychologists and coaching staff working in elite women's rugby to consider. While striving to enhance social identity among players to improve sporting performance, equal measures should be put in place to ensure that the identity players share does not adversely affect player health and wellbeing, and their injury management and disclosure of SRC. Open and transparent communication between all involved in player injury is also essential to avoid players left feeling isolated and confused.

### 4.5. Conclusion

This study explored the influence of social identity on SRC disclosure in elite women's rugby. Our research has uniquely identified a number of mediators that could be further investigated to ensure that women in rugby are predisposed to disclose SRC symptomology. Encouragingly players’ shared social identity can be utilised to enhance and reinforce SRC education messages. Practitioners should explore how players can be incorporated into coaching and medical teams to encourage engagement with SRC education interventions and to improve communication and trust.

## Data Availability

The original contributions presented in the study are included in the article/Supplementary Material, further inquiries can be directed to the corresponding author.

## Appendix

### Interview guide—semi-structured

#### Section A: introduction

1. General background: name, location, brief summary of career (start to finish)
2. Sequence/details of career up to present day per club
3. Duration of career
4. What made you decide to go into/get involved with elite/semi-elite women's rugby/kept you involved?
5. Pros and cons of professional rugby (subjective)
6. Injuries experienced/condition of your body currently/long term effects of a rugby career?
7. What injuries were you most worried about sustaining as a player?

#### Section B: knowledge

1. How would you describe a concussion to another person?
2. Have you experienced a concussion?* (some questions below may not be relevant if not). (*Researcher to state all symptoms and repeat Q2 if needed—Based on what we have discussed, do you now think you may have had a concussion)*
3. Concussion awareness while playing/concussion incidence while playing.
4. Did concussion awareness change throughout your playing career?
5. What type of symptoms would you say are typically associated with concussions?
6. Would you or did you continue playing a sport while having a headache that resulted from a suspected concussion? Or did you see others do this?
7. Did you feel a responsibility to return to a game even if it meant playing while still experiencing symptoms of concussion? (*or again refer to others they knew if not themselves*)
8. Are concussions considered less important than other injuries in your opinion?
9. Do you think having one concussion means you are more likely to have another concussion?
10. How did you see players diagnosed with a concussion, do you have to be knocked out?
11. Did you see instances when players clearly had a concussion on the field but were not removed from play?
12. Have you seen any one experience a concussion where there isn't a direct hit to the head?
13. How long do you think/did your symptoms of a concussion last (*several weeks or gone after 10 days—prompt don't say*)
14. Do you think brain imaging will show physical damage after a concussion? Post-concussion? (*brain imaging (CAT Scan, MRI, X-Ray, etc.) typically shows visible physical damage (e.g. bruise, blood clot) to the brain)*
15. Did you or a fellow player experience emotional disruptions post impact/concussion? (*can personalise if necessary)*
16. What is your personal attitude to concussion, do you think it is overhyped? (*Personal attitude towards concussion as a player)*

#### Section C: management, treatment, support (post-concussion)

1. What was the initial on field management (i.e., identification and removal)—was it consistent across clubs?
2. Were there aligned protocols across the organisation (i.e. coaching, medical, S&C)? (*Lines of communication with medical teams/health professionals*)
3. Were coaches cautious when determining whether an athlete should return to play (RTP), did coaches dictate RTP?
4. Personal experience on return to play protocols—variance between amateur and/or elite/semi-elite (positives and negatives)
5. Personal approach to recovery from impacts/concussion
6. Do you think there is a risk to long-term health and wellbeing from multiple concussions?
7. Did you experience any impediments to recovery and barriers to treatment and recovery length?
8. Did you have support from other sources (i.e., neuroscientists, etc.)?
9. Do you think any sport equipment, e.g., mouthguards or soft-shell helmets, protect the brain and/or being knocked out?

### Conclusions

1. What law changes do you think could be introduced to reduce injury risk and/or concussion risk (*Follow on—do you think they should be introduced*?)
2. Can you suggest any changes to how the game is played to mitigate (any) injury concussion risk?
3. Can coaches, club owners do anything to reduce injury risk or long-term effects of injury on retired players? Do you think this is their responsibility?
4. Closing comments that you’d like to include
5. Thanks and next steps.
